# *Drymaria veliziae* (Caryophyllaceae), a new species from the Andes of Cajamarca (North Peru)

**DOI:** 10.3897/phytokeys.140.47738

**Published:** 2020-02-24

**Authors:** Daniel B. Montesinos-Tubée, Carolina Tovar, Gustavo Iberico-Vela, Juan Montoya-Quino, Isidoro Sanchez-Vega

**Affiliations:** 1 Naturalis Biodiversity Centre, Darwinweg 2, 2333 CR Leiden, The Netherlands Naturalis Biodiversity Centre Leiden Netherlands; 2 Instituto Científico Michael Owen Dillon, Av. Jorge Chávez 610, Cercado, Arequipa, Perú Instituto Científico Michael Owen Dillon Arequipa Peru; 3 Instituto de Ciencia y Gestión Ambiental de la Universidad Nacional de San Agustín de Arequipa, Calle San Agustín 108, Arequipa-Perú Universidad Nacional de San Agustín de Arequipa Arequipa Peru; 4 Royal Botanic Gardens, Kew, The Jodrell Laboratory, Royal Botanic Gardens, Kew, Surrey TW9 3DS, UK Royal Botanic Gardens Surrey United Kingdom; 5 Universidad Nacional de Cajamarca, Herbario CPUN, Departamento de Biología, Cajamarca, Perú Universidad Nacional de Cajamarca Cajamarca Peru

**Keywords:** Andes, Cajamarca, new species, Caryophyllaceae

## Abstract

A new species from the Northern Peruvian Andes (Cajamarca department), *Drymaria veliziae***sp. nov.**, is proposed in the present paper. It grows in the high-elevation montane grasslands and it is morphologically similar to *D. auriculipetala* from which it differs in having elliptic-ovate leaves, blade margin bases glandular, large number of stipules arranged in a pedicel form at the leaf axis and by the short and glandular pedicels. A detailed description, original photographs and a location map are provided, as well as an updated diagnostic key of Drymaria Ser. Frutescens. The IUCN status of the new species is assessed as Endangered (EN).

## Introduction

The genus *Drymaria* Willd. ex Schult. (Caryophyllaceae Juss.) contains 48 species mainly distributed in subtropical regions of the Western Hemisphere (see the most recent revision of the genus by Duke 1961), whereas one species (*Drymaria cordata* Willd. ex Schult.) is widespread, occurring in Asia, Africa, Oceania, and Madagascar (Villarreal and Estrada 2008). Duke (1961) recognised 17 Series but they were not validly published because Latin diagnoses were not given (*nomina nuda*; see Art. 38.2 Ex. 1 of ICN, Turland et al. 2018) (see Hartman 2005 and Villarreal and Estrada 2008). After Duke (l.c.), no studies have been made on Peruvian *Drymaria* taxa.

Concerning the molecular data, those available for *Drymaria* are included in the large phylogenetic study of Caryophillaceae by Greenberg and Donoghue (2011), but here no Andean species of *Drymaria* were involved.

On the basis of some authors (Macbride 1937; Brako and Zarucchi 1993) and our ongoing studies (Montesinos-Tubée in prep.), 24 *Drymaria* species (including 18 infraspecific taxa) are expected to occur in the Peruvian Andes.

As part of the ongoing floristic and taxonomic studies on Peruvian Flora (Montesinos-Tubée 2013; Montesinos-Tubée and Kool 2015; Montesinos-Tubée et al. 2018), we found an interesting population belonging to the genus *Drymaria* which, however, cannot be identified with any of the currently known species. We, therefore, decided to propose a new species for Science.

## Material and methods

Specimens of *Drymaria*, housed in many South American and other herbaria (B, CUZ, F, HSP, HUT, HUSA, K, L, LP, LPB, MOL, P, SI, SGO, USM, WAG; acronyms according to Thiers 2019+), were studied by the first author (DBM-T). Additionally, field surveys were carried out. Specimen information (including digital images) were searched using online sources such as GBIF (2019), JSTOR Global Plants (2019), Tropicos (2019) and herbarium databases of several herbaria.

Morphological characters were studied using a NSZ-405 1X-4.5X stereomicroscope and an AmScope M100CLED 40×-1000× compound microscope. Conservation assessments were undertaken using the IUCN Red List Criteria (IUCN 2019). The monograph by Duke (1961) was used as the basic reference to describe the new species.

## Results and discussion

### Drymaria veliziae

Taxon classificationPlantaeCaryophyllalesCaryophyllaceae

Montesinos sp. nov.

CB4C39AD-D748-5373-AAC1-513FDA856B0D

urn:lsid:ipni.org:names:77206319-1

#### Type.

Peru. Cajamarca: Cajamarca: Encañada: Chanta baja, on sandy clay loam soils amongst shrub species and tussock grasslands, close to agricultural lands, 3295 m elev., slope of 60% and rock cover of 35%, 6°49’56’’S, 78°30’20’’W (DMS). 06 June 2009, *C. Tovar 1058* (holotype CPUN–22705!).

#### Diagnosis.

*Drymaria veliziae* is similar to *D. auriculipetala* Mattf. from which it differs in having glands covering the stems and pedicels, leaves with elliptic-ovate form, shorter in size (4–5.5 mm vs. 5–15 mm in *D. auriculipetala*), by the leaves arranged in fascicules (vs. simple opposite leaves), stipules in numbers of 14–20 per axis (vs. 2–4 in *D. auriculipetala*), pedicel size (1–2 mm long vs. 5–40 mm) and by the capsule size being smaller (1.4–1.6 mm vs. 3–4 mm).

**Figure 1.**
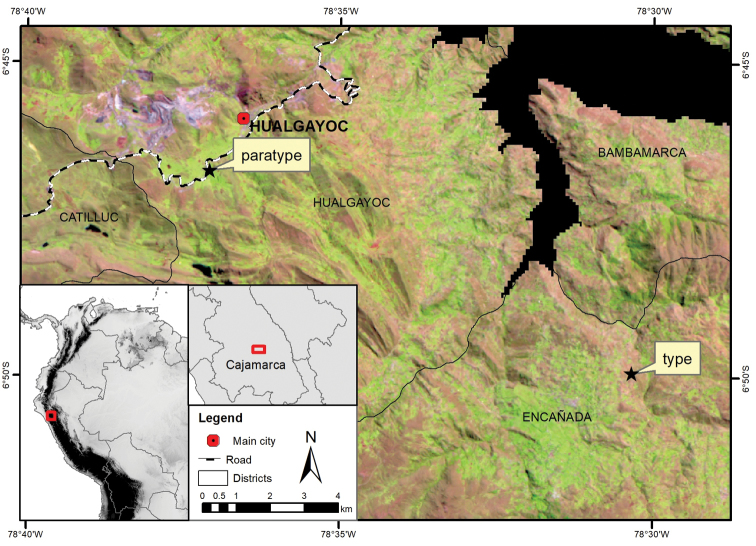
Location map of the type and paratype collection localities. Background: LANDSAT 5TM satellite image (June 2007) where light brown-pink areas represent Jalca grasslands, light green areas are agricultural fields and purple represents mining areas.

#### Description.

Perennial herb, the taproot woody, stems originating from the root brow spreading or ascending, rarely decumbent, of 20–35(–50) cm long. Stems rigid, greenish-lilac, densely glandular, of about 0.05–0.3 mm long, persistent on mature stems and having scattered plicate trichomes of 1–3 mm long on young stems. Internodes 0.2–5.5 cm long. Leaves opposite, usually forming short fascicules; petioles 0.3–0.9 mm long, partially glaucous, scarcely covered with glands in the margins; blades elliptic to ovate, 4–5.5 mm long × 1.2–2 mm width, coriaceous, the bases cuneate, decurrent to the petiole, the apex aristate, 1–1.5 mm long, narrowly bearing short glands along the margin, midrib nearly inconspicuous; leaf margins lustrous, revolute and glabrous except at the base; stipules aciculate to linear-lanceolate, aristate, 1.5–3 mm long × 0.1–0.4 wide, shorter or equalling the length of the leaves, with glabrous margins and usually verticillate, in numbers of 14–20 per axis, persistent, white translucid to brownish with age, rarely bifid or trifid; bracts opposite, 2.5–3 mm long × 2 mm width, involute, cupuliform, irregularly ovate, margins covered with scattered glands, surface white coloured with lilac blotches. Flowers except the first formed, axilar and solitary at the end of the branches, base protected by the bracts (in pairs 1 or 2). Pedicels 1–2 mm long, densely glandular and covered with carinate plicate trichomes, rarely aereal, of about 0.2–0.4 mm. Calyx cylindrical-campanulate; sepals 5, equal, 5–6 mm long × 2–2.2 mm width, glabrous, elliptic-ovate, apex apiculate and aristate, basally truncate, 3–5 nerved; petals 5, 5–7 mm long × 1.8–2.2 mm width, bifid about half their length, elliptic, apex rounded, 1–1.2 mm width, 8–10 nerved, constricted at the junction of the lobes; stamens 5, 2–2.2 mm long, anthers oblong, 0.2–0.3 mm long; ovary roundish, 1–1.4 mm long, slightly exceeded by the anthers; style 1–1.2 mm long, trifid about half its length, stigmatic branches twisted or coiled. Capsule ovoid, 1.4–1.6 mm long, 5–8 seeded. Seeds roundish, reniform, 0.6–0.9 mm long × 0.6–0.8 mm wide, granulate, ventral surface with roundish-acute tubercles, black to dark brown in colour.

**Figure 2.**
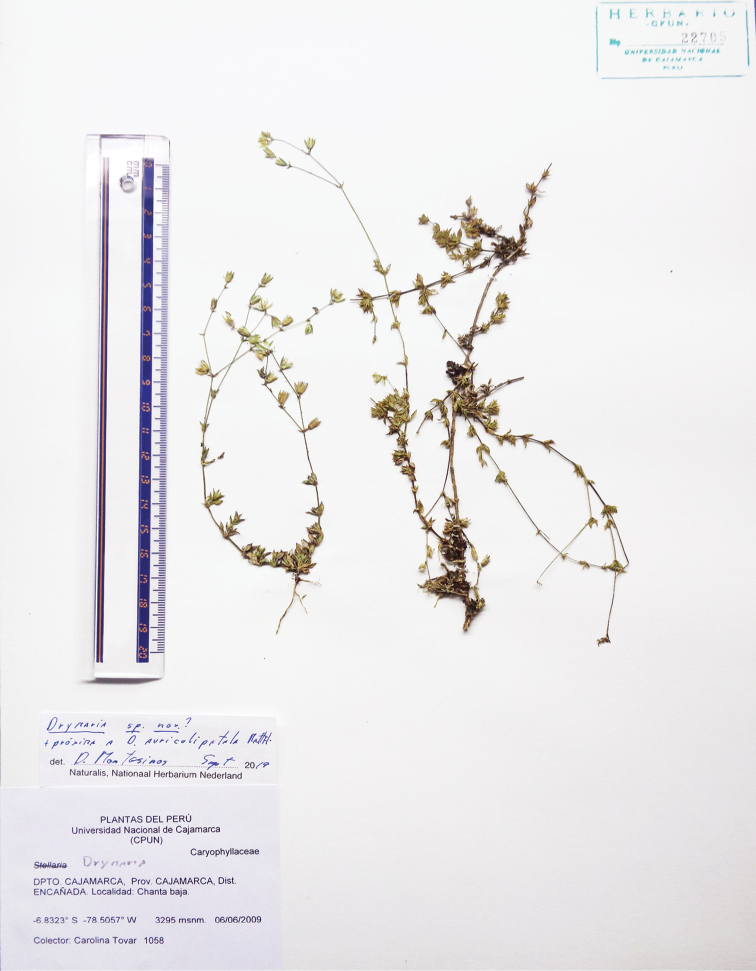
Holotype of *Drymaria veliziae* (CPUN-22705!).

**Figure 3.**
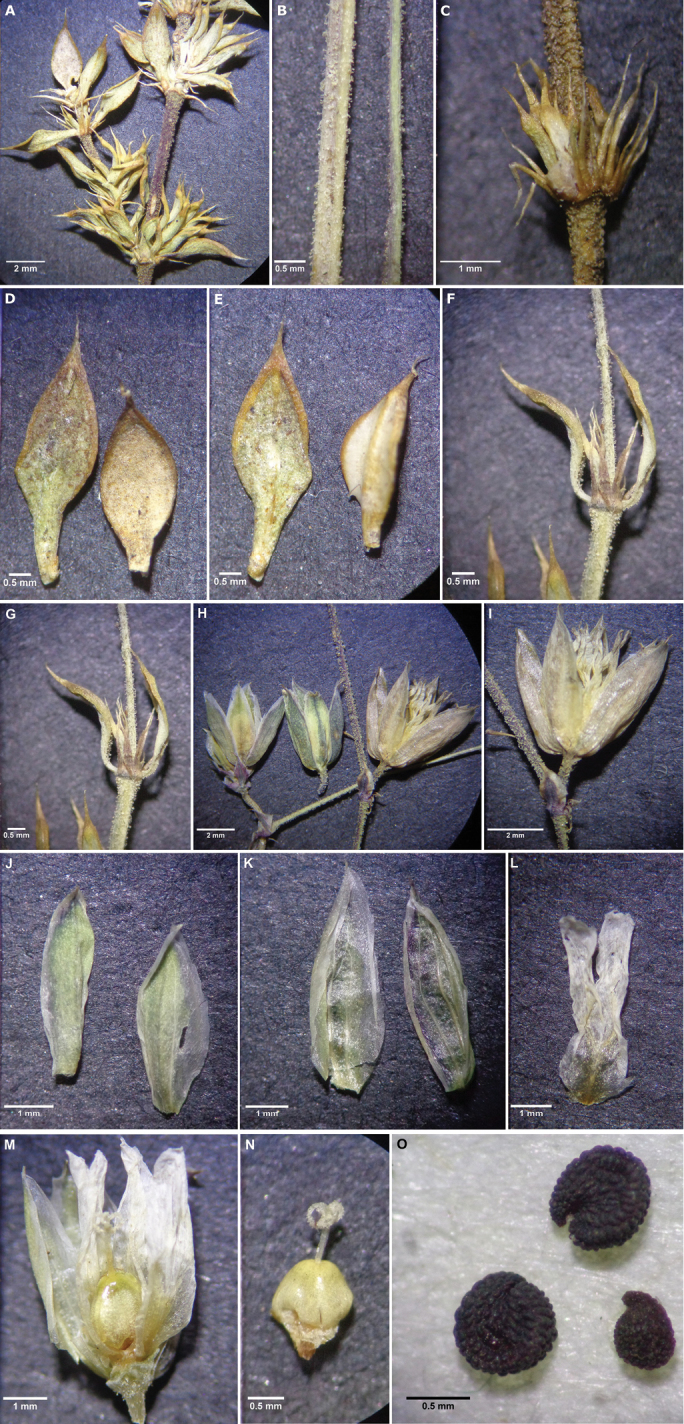
A. Internode and leaf arrangements **B** glandular stems **C** crown of stipules at the leaf axis **D** leaves (adaxial side) **E** leaves (abaxial side) **F** axis of leaves **G** bracts at the leaf axis **H** mature and immature flowers **I** flower detail **J** sepals (adaxial side) **K** sepals (abaxial side) **L** single petal with bifid apex portion **M** flower with ovary, stamens (indicated with dark lines) and style **N** ovary detail with a trifurcated style **O** seeds.

#### Etymology.

The epithet “*veliziae*” honours Claudia Véliz Rosas (1978–2019), a passionate biologist who devoted her research efforts to the study Peruvian biodiversity. Her deep love of nature, people and travelling inspired her to work throughout Peru, studying freshwater, marine and mountain ecosystems. Her research contributed to the establishment of protected areas and the development of management plans. Claudia dedicated many years to study taricaya turtles in the Amazon, helping local human communities to improve taricayas’ management and conservation. She was an excellent and supportive friend, a talented amateur painter and dancer and a keen cyclist.

**Paratype**: Peru: Cajamarca: Hualgayoc: Hualgayoc, less than 1 km from the Goldfield mine, surrounded by agricultural fields downslope, found on sandy clay loam soils, 3715 m elev., 6°46’43’’S (DMS) and 78°37’5’’W (DMS), 100% slope and 5% rock cover. 01 June 2009, *C. Tovar 909* (CPUN–22858!).

**Figure 4.**
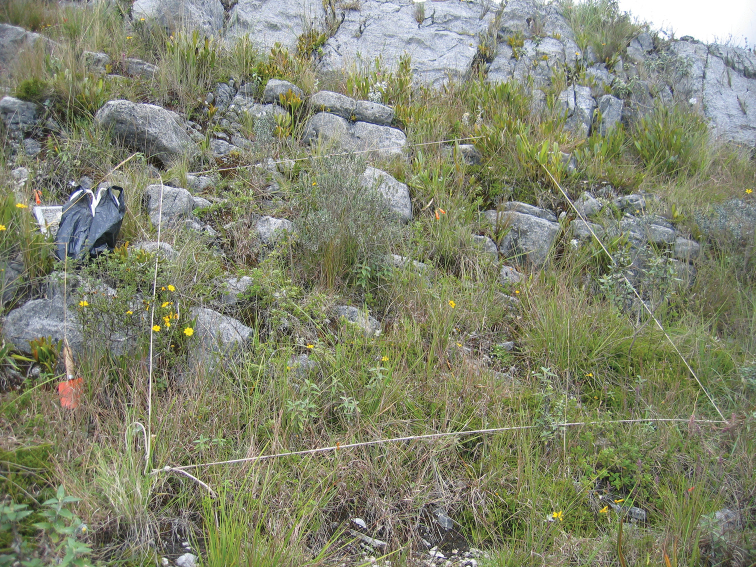
Quadrat where the new species was found. Chanta baja, Encañada District, 3295 m elev. Photo by Carolina Tovar.

**Ecology and distribution**: *Drymaria veliziae* grows on steep mountain cliffs (slope 60–100%) on sandy clay loam soils at an elevation of 3295–3715 m on the eastern slopes of the Jalca, on the headwaters of the Llaucano River, tributary of the Marañon River. Climatic characteristics for the localities of the type and paratype, extracted from the CHELSA climatology (Karger et al. 2017), show mean annual temperatures in these areas are 8.5–11.5°C, with minimum temperatures estimated between 1.8 and 5°C. Total annual precipitation ranges from 900 to 1200 mm with driest months receiving 16–28 mm. Other species found in the two localities were *Hieracium peruanum* Fr. (Asteraceae), *Hypochaeris taraxacoides* (Meyen & Walp.) Ball (Asteraceae) and *Calamagrostis* spp. (Poaceae). In the type locality of *Drymaria veliziae* (Fig. 4), it has been observed that it grows associated with shrubs (e.g. *Coreopsis senaria* S.F. Blake & Sherff (Asteraceae), *Achyrocline celosioides* (Kunth) DC. (Asteraceae), *Ageratina cutervensis* (Hieron.) R.M. King & H. Rob. (Asteraceae)), tussock grasses (*Calamagrostis* and *Festuca* spp. (Poaceae)) and an orchid (e.g. *Masdevallia* spp.), amongst others. The locality of the paratype, being at higher altitude, had less species richness and associated species with *D. velizzii* were *Geranium peruvianum* Hieron. (Geraniaceae), *Calceolaria concava* Molau (Calceolariaceae), *Chrysactinium acaule* (Kunth) Wedd. (Asteraceae), *Euphorbia huanchahana* (Klotzsch & Garcke) Boiss. (Euphorbiaceae), amongst others.

#### Taxonomical notes.

On the basis of the classification proposed by Duke (1961), *Drymaria veliziae* would belong to the ser. Frutescens Duke sharing the leaf shape (linear to lanceolate) glandular pedicels, the number of sepal nervadures (3–5) and petals bifid which are not tapered to the claw.

*Drymaria veliziae* is morphologically similar to *D. auriculipetala* Mattf. (1936: 438–439) but differs in having glands covering the stems and pedicels, leaves with elliptic-ovate form, shorter in size, by the leaves arranged in fascicules (vs. simple opposite leaves), stipules larger numbers per axis, shorter pedicel size and smaller capsule size.

Furthermore, *Drymaria veliziae* differs from *D. stereophylla* Mattf. (1936: 436–437) by the plant habit, the glabrous surface of the leaves (vs. presence of glands and puberulent trichomes in *D. stereophylla*), bifid or trifid stipules (vs. entire), shorter stamen size (2–2.2 mm vs. 4–6 mm), shorter style size (1–1.2 mm vs. 1–2.5 mm), capsule size shorter (1.4–1.6 vs. 2.5–3.5 mm) and seed size (0.6–0.9 vs. 0.9–1.3 mm in *D. stereophylla*).

The new species is further differentiated from *D. stellarioides* Willd. ex Schult. (1819: 406) by the stipule form (bifid to trifid vs. entire), shorter bract size (2.5–3 mm vs. 3–5 mm in *D. stellarioides*), sepals glabrous (vs. glabrous to densely glandular-puberulent) and shorter capsule size and form (1.4–1.6 mm, ovoid vs. 3–5 mm long, ellipsoid).

An updating of the diagnostic key for the ser. Frutescens, as proposed by Duke (1961: 214) follows:

**Table:** 

1	Pedicels and sepals present and glabrous, sepals (3-)5-nerved	**2**
–	edicels and sepals present or absent, glabrous or glandular, sepals 3-nerved	**3**
2	Leaves imbricate, closely appressed to the stems, 2–6 mm long, 1–1.5 mm broad, basally clasping and pungently acute; the sepals mostly 5-nerved; petals tapered to the claw	***D. frutescens***
–	Leaves not imbricate, 4–12 mm long, 2–6 mm broad, apically acute to aristately acuminate; the sepals 3–4-nerved; petals not tapered to the claw	***D. stereophylla***
3	Pedicels and sepals usually glandular, leaves glabrous, apically aristate-attenuate, aristate and basally cuneate; stipules present; seeds with domical or conical tubercles	**4**
–	Pedicels and sepals glabrous to densely glandular-puberulent; leaves apically acute and marginally entire, densely glandular-puberulent; stipules entire, apparently fused or occasionally absent; seeds without domical tubercles	***D. stellarioides***
4	Bracts absent, simple opposite leaves, pedicels 5–40 mm long, stipules copiously shorter than the leaves; glabrous pedicels and stems, short number of pedicels per axis (2–4)	***D. auriculipetala***
–	Bracts present; leaves arranged in fascicules; pedicels 1–2 mm long, glands covering the stems and pedicels; leaves with elliptic-ovate form, large number of pedicels per axis (14–20)	***D. veliziae***

#### Conservation status.

Only the two localities referring to holotype and paratype are currently known for *Drymaria veliziae* (these localities are separated by about 12 km). The surrounding areas are characterised by various types of human activities, for example, agriculture, land conversion, forestry with exotic species, slash burning, natural resource extraction, amongst others (Figure 1). Land use change occurred between 1987 and 2007 with a reduction of the 25% of grasslands and an increasing of landscape fragmentation (see Tovar et al. 2013). The type specimen was collected on a Jalca patch surrounded by agricultural fields (*Vicia faba* L. (Fabaceae), *Solanum tuberosum* L. (Solanaceae), *Zea mays* L. (Poaceae)), while the paratype was collected in a smaller patch less than 1 km distant from a mining area developed after 1987. A total of 110 vegetation plots were sampled across the Jalca in 2007 (Tovar et al. 2012) and the new species was found in only two of them. Using the criteria B1a and B1b of the IUCN (2019), we assessed *D. veliziae* as Endangered species (EN).

## Supplementary Material

XML Treatment for Drymaria veliziae

## References

[B1] Brako L Zarucchi J (1993) Catalogue of the Flowering Plants and Gymnosperms of Peru. Monographs in Systematic Botany from the Missouri Botanical Garden 45: 1–1286.

[B2] Duke JA (1961) Preliminary revision of the genus *Drymaria*. Annals of the Missouri Botanical Garden 48(3): 173–268. 10.2307/2394953

[B3] GBIF (2019) Global Biodiversity Information Facility. https://www.gbif.org/es/ [accessed: 10 December 2019]

[B4] Greenberg AK Donoghue MJ (2011) Molecular systematics and character evolution in Caryophyllaceae. Taxon 60(6): 1637–1652. 10.1002/tax.606009

[B5] Hartman RL (2005) *Drymaria*. In: Flora of North America Editorial Committee (Eds) 1993+.Flora of North America North of Mexico 12+ vols. New York and Oxford, vol. 5, 9–14.

[B6] IUCN (2019) The IUCN Red List of Threatened Species. Version 2019-2. http://www.iucnredlist.org [accessed: 18 July 2019]

[B7] Global Plants JSTOR (2019) JSTOR Global Plants. http://plants.jstor.org/ [accessed: 15 October 2019]

[B8] Karger DN Conrad O Bohner J Kawohl T Kreft H Soria-Auza RW Zimmermann N Linder HP Kessler M (2017) Climatologies at high resolution for the earth’s land surface areas. Scientific Data 4(1): 170122 10.1038/sdata.2017.122 28872642 PMC5584396

[B9] Macbride JF (1937) Caryophyllaceae, Flora of Peru. Publications of Field Museum of Natural History, Botanical Series 13: 617–626.

[B10] Mattfeld J (1936) Einige neue Drymaria-Arten aus Peru. Notizblatt des Königl. Botanischen Gartens und Museums zu Berlin 13(118): 436–444. 10.2307/3995022

[B11] Montesinos-Tubée DB (2013) Paronychia ubinensis (Caryophyllaceae: Paronychioideae) a new species from Moquegua, South Peru. Phytotaxa 124(1): 50–54. 10.11646/phytotaxa.124.1.6

[B12] Montesinos-Tubée DB Kool A (2015) Arenaria acaulis (Caryophyllaceae), a new species from South Peru. Phytotaxa 220(1): 77–82. 10.11646/phytotaxa.220.1.7

[B13] Montesinos-Tubée DB Cano A García-Llatas L Ju Y Kool A (2018) *Paronychia sanchez-vegae* (Caryophyllaceae), a new woody species of *Paronychia* from North Peru. Phytotaxa 334(1): 41–48. 10.11646/phytotaxa.334.1.6

[B14] Schultes JA (1819) Caroli a Linné equitis Systema Vegetabilium secundum Classes, Ordenes, Genera, Species cum characteribus, dipferentiis et synonimiis. Stuttgardtiae 5: 406.

[B15] Thiers B (2016) Index Herbariorum: A global directory of public herbaria and associated staff. New York Botanical Garden's Virtual Herbarium. http://sweetgum.nybg.org/ih

[B16] Tovar C Duivenvoorden JF Sánchez-Vega I Seijmonsbergen AH (2012) Recent changes in patch characteristics and plant communities in the Jalca grasslands of the Peruvian Andes. Biotropica 44(3): 321–330. 10.1111/j.1744-7429.2011.00820.x

[B17] Tovar C Seijmonsbergen AC Duivenvoorden JF (2013) Monitoring land use and land cover change in mountain regions: An example in the Jalca grasslands of the Peruvian Andes. Landscape and Urban Planning 112: 40–49. 10.1016/j.landurbplan.2012.12.003

[B18] Tropicos (2019) Tropicos.org. Missouri Botanical Garden. Available from: http://www.tropicos.org/ [accessed: 10 October 2019]

[B19] Turland NJ Wiersema JH Barrie FR Greuter W Hawksworth DL Herendeen PS Knapp S Kusber W-H Li D-Z Marhold K May TW McNeill J Monro AM Prado J Price MJ Smith GF (2018) International Code of Nomenclature for algae, fungi, and plants (Shenzhen Code) adopted by the Nineteenth International Botanical Congress Shenzhen, China, July 2017. Koeltz Botanical Books. 10.12705/Code.2018

[B20] Villarreal JA Estrada AE (2008) A new species of *Drymaria* (Caryophyllaceae) from northeastern Mexico. Brittonia 60(4): 329–331. 10.1007/s12228-008-9028-x

